# Psychometric properties of a condition-specific PROM for the psychosocial consequences of Labelling hypertension by using Rasch analysis

**DOI:** 10.1186/s41687-021-00291-4

**Published:** 2021-02-04

**Authors:** János Valery Gyuricza, Karl Bang Christensen, Ana Flávia Pires Lucas d’Oliveira, John Brodersen

**Affiliations:** 1 Departamento de Medicina Preventiva, Faculdade de Medicina da Universidade de São Paulo, Av. Dr Arnaldo, 455 2 andar. CEP, São Paulo, SP 01246-903 Brazil; 2 Department of Public Health, Section of General Practice and Research Unit for General Practice, University of Copenhagen, Øster Farimagsgade 5, building 24, P.O. Box 2099, 1014 Copenhagen K, Denmark; 3 Department of Public Health, Section of Biostatistics, University of Copenhagen, Øster Farimagsgade 5, Building 15, P.O. Box 2099, 1014 København K, Denmark; 4 Primary Health Care Research Unit, Copenhagen, Region Zealand Denmark

**Keywords:** Hypertension, Psychosocial consequences, Patient-reported outcome, Psychometric properties

## Abstract

**Background:**

A previous qualitative assessment of the psychosocial consequences of labelling hypertension describes the diagnosis of hypertension as a labelling event with potential unintended negative long-term psychosocial consequences (labelling effects). Until now, the benefits of diagnosing hypertension have been far more reported than the harms. To obtain the net result of the preventive interventions for cardiovascular disease, such as diagnosing and treating mild hypertension, assessing benefits and harms in the most comprehensive way possible is necessary, including the psychosocial consequences of labelling. When measuring psychosocial consequences of labelling hypertension, a questionnaire with high content validity and adequate psychometric properties is needed.

**Objectives:**

The aim of this study was to describe the psychometric parameters of face and content-validated pool of items. Other objectives were also to screen the item pool by using Rasch model analysis and confirmatory factor analysis (CFA) for identifying such items with sufficient fit to the hypothesised models.

**Methods:**

We surveyed the pool of items as a draft questionnaire to Brazilians recruited via social networks, sending e-mails, WhatsApp® messages and posting on Facebook®. The inclusion criteria were to be older than 18 years old, to be healthy and to have only hypertension.

We used Rasch model analysis to screen the item pool, discarding items that did not fit the hypothesised domain. We searched for local dependence and differential item functioning. We used CFA to confirm the derived measurement models and complementarily assessed reliability using Cronbach’s coefficient alpha.

**Results:**

The validation sample consisted of 798 respondents. All 798 respondents completed Part I, whereas 285 (35.7%)—those with hypertension—completed Part II. A condition-specific questionnaire with high content validity and adequate psychometric properties was developed for people labelled with hypertension. This measure is called ‘Consequences of Labelling Hypertension Questionnaire’ and covers the psychosocial consequences of labelling hypertension in two parts, encompassing a total of 71 items in 15 subscales and 11 single items.

**Conclusion:**

We developed a tool that can be used in future research involving hypertension, especially in scenarios of screening, prevention, population strategies and in intervention studies. Future use and testing of the questionnaire may still be required.

**Supplementary Information:**

The online version contains supplementary material available at 10.1186/s41687-021-00291-4.

## Background

Approximately one-fourth of the world’s population has blood pressure above the diagnostic threshold for hypertension [1]. Among them, the lowest risk group is that with mild hypertension, which accounts for roughly 60% of the people diagnosed with hypertension Preventive population strategies may reduce cardiovascular disease (CVD) burden [2]. However, previous studies failed to prove the benefits of the primary prevention of CVD on the basis of a risk strategy—pharmacologic treatment—for people with mild hypertension [3].

To obtain the net result of preventive interventions for CVD, such as diagnosing and treating mild hypertension, assessing the benefits and harms in the most comprehensive way possible is wise. Until now, studies seem to overlook all possible harms; specifically, psychosocial harms have been far less studied than potential benefits [4].

One unintended harm that has been recognised but has not been comprehensively studied are the negative psychosocial consequences labelling hypertension. Sir George Pickering suggested that hypertension labelling may evoke a feeling of fear of the affliction of a serious disease in a patient [5]. In the next decades, this issue has been addressed in a few studies [6]. One seminal study among Canadian steelworkers [7, 8] suggests that after the diagnosis of hypertension, a few negative psychosocial consequences are observed: people experience additional symptoms, increase in absenteeism, become dependent on the healthcare system, worsen their marital relations and are psychologically distressed. The same effects are not observed in those that are unaware of their diagnosis. This study provided relevant insights into the extension of the possible negative effects of labelling but failed to obtain patient-reported outcomes and failed to fully uncover the psychosocial consequences of labelling hypertension. Patient-reported outcomes are reports that come directly from patients about the status of their health condition without the amendment of interpretation of their response by an interviewer [9] and are considered prerequisites for the assessment of psychosocial consequences [10].

A previous qualitative assessment of the psychosocial consequences of labelling hypertension described the diagnosis of hypertension as a labelling event with potential unintended negative long-term psychosocial consequences (here assumed to be the same as negative effects of labelling) [11]. Similar results are confirmed by our research group in our study population [12].

Haynes et al. recently conducted a large study and found an elevated risk of psychological distress in people aware of the diagnosis of hypertension [13]. However, Haynes used the generic measure GHQ-12. The GHQ-12 is a self-administered screening questionnaire, designed for use in consulting settings aimed at detecting those with a diagnosable psychiatric disorder [14]. The purpose of GHQ-12 is not the same as measuring the psychosocial consequences of labelling hypertension.

Furthermore, the use of short-form-12 (SF-12) and SF-36 [15] was also proposed to assess the consequences related to hypertension. However, SF-12 and SF-36 are self-administered generic measures for health-related quality of life, and may also fail to measure the psychosocial consequences of labelling hypertension.

Generic measures are instruments designed to be used in broad variety of contexts and to be applicable across conditions and interventions [16]. The downside is that generic measures may lack content validity (coverage and relevance) in terms of specific conditions [10]. Studies have also shown inconsistent psychometric properties of generic instruments when used across different populations [17, 18]. Finally, the use of generic measures becomes problematic if the people who fare least well are also those who find the generic instruments of least relevance [19].

The alternative is to use condition-specific measures, which are instruments that focus on health aspects that are relevant to a specific group of people. Condition-specific instruments are more sensitive and insure higher content coverage than generic measures [16].

The psychosocial consequences of labelling hypertension seem to be a remarkably frequent patient-reported condition-specific harm [4], and a new questionnaire with high content validity and adequate psychometric properties is needed [10]. Methods, which allow accurate measurements of constructs, such as the psychosocial consequences of labelling hypertension, have been developed [20]. One of them is the combination of patient-reported outcomes [9] and Rasch model analysis [10, 21–23]. In this combination, the development of patient-reported items from qualitative interviews can support the relevance and coverage of the items (content validation) and group the items in different hypothesised domains related to a latent variable. Rasch model analysis can help with determining whether the items grouped in a domain are appropriate indicators and can measure different nuances of the hypothesised latent variable. Such an evidence is necessary to be able to postulate that the score of each item can be added in a sum-score of all the items in a unidimensional scale [24]. Moreover, confirmatory factor analysis (CFA) can be used to confirm the findings of the Rasch model analysis.

Therefore, the aim of this study is to use Rasch model analysis and CFA to screen the pool of items, identify items with sufficient fit to the model and describe the psychometric parameters of the final pool of items.

The tool is not designed to be used in a clinical setting with individual patients to answer questions, such as ‘Is my patient experiencing harms of being labelled?’ The purpose of this tool is to allow for the measurement of the psychosocial consequences of labelling hypertension in groups of patients and populations. Such measurement is relevant because it can include previously unmeasured harms of being labelled with the diagnosis of hypertension, which can be included in the assessment of the balance between the benefits and harms of medical interventions for preventing CVD in screening for hypertension and for cardiovascular risk assessment [9]. This is a patient-reported outcome measure (PROM) and is supposed to assess the psychosocial consequences of labelling hypertension more accurately than previous measures with GHQ-12, SF-12 and SF-36.

## Methods

We previously developed a Brazilian Portuguese pool of items aiming for the psychosocial consequences of labelling hypertension (Table 1) [12]. That is, we translated items from all versions of the Danish Consequences of Screening (COS) questionnaires [25–28] to Brazilian Portuguese. These COS items, which can be found in all versions of COS, were called ‘core’ items. Those found in specific versions of COS were called ‘disease-specific’ items. Then, we conducted single and group interviews with people with hypertension who had low risk of CVD. Subjects selected for our qualitative research had to have a clinically confirmed diagnosis of hypertension with the prescription of antihypertensive medication; we also included only those without comorbidities. These interviews had three main objectives: to test translated items for face and content validity, generate new relevant items to achieve high content validity and to categorise the new items in domains. The items generated on the basis of the interviews were called ‘new’ items. High content validity was achieved. The result was a set of 132 items divided into 22 domains in two parts. Part I was directed at the general population and encompassed 84 items in 14 domains and 10 single items. Part II was directed only at people diagnosed with hypertension and encompassed 36 items in eight domains and two single items. All items, domains and parts are presented in Table 1. With these methods, we established content relevance and content coverage among Brazilians. To our best knowledge, no other PROM has been developed for the consequences of labelling hypertension.

**Table 1:** Item pool

**Part**	**Item number**	**Included in final item set?**	**Questionnaire of origin**	**Domain**	**Brazilian Portuguese version**	**English ad hoc translation**
I	2	NO	core	Anxiety	Me senti preocupado com meu futuro	I felt worried about my future
I	3	NO	core	Anxiety	Me senti amedrontado	I felt frightened
I	4	NO	core	Anxiety	Me senti com medo	I felt scared
I	13	YES	core	Anxiety	Me senti emotionalmente fora do meu normal	I felt emotionally out of my normal
I	14	YES	core	Anxiety	Me senti inquieto	I felt restless
I	15	YES	core	Anxiety	Me senti nervoso	I felt nervous
I	16	YES	core	Anxiety	Me senti ansioso	I felt anxious
I	25	YES	core	Anxiety	Me senti a ponto de entrar em pânico	I felt about to panic
I	29	YES	disease specific	Anxiety	Me senti em estado de choque	I felt in shock
I	61	NO	new	Anxiety	Me senti impaciente	I felt impatient
I	93	NO	core	Anxiety	Me senti agitado	I felt agitated
I	5	YES	core	Behaviour	Me senti irritado	I felt annoyed
I	6	NO	core	Behaviour	Me senti mais quieto do que o normal	I felt more quiet than usual
I	9	NO	core	Behaviour	Me senti com dificuldade de me concentrar	I felt hard to concentrate
I	11	NO	core	Behaviour	Tive mudanças em meu apetite	I had changes in my appetite
I	18	NO	core	Behaviour	Me senti mais fechado	I felt introverted
I	22	YES	core	Behaviour	Tive dificuldades em realizar meu trabalho e outras tarefas semelhantes	I had difficulties doing my job and other similar tasks
I	24	YES	core	Behaviour	Tive dificuldades em realizar tarefas de casa	I had difficulties doing domestic work
I	30	NO	new	Blood pressure related	Fiquei com medo da pressão alta o tempo todo na cabeça	I had the fear of high blood pressure all of the time in the head
I	57	NO	new	Blood pressure related	Pensei que seria melhor se não soubesse que tenho pressão alta	I thought it would be better if I didn’t know I have high blood pressure
I	90	NO	new	Blood pressure related	Tive sintomas de pressão alta	I had symptoms of high blood pressure
I	37	YES	disease specific	Body Perception	Me senti doente	I felt sick
I	38	YES	disease specific	Body Perception	Tive a sensação de que havia algo errado com meu corpo	I had a feeling something was wrong with my body
I	42	NO	disease specific	Body Perception	Me senti como se meu corpo fosse uma máquina que não funciona	I felt like my body was a non-working machine
I	46	YES	disease specific	Body Perception	Me senti como um estranho em meu próprio corpo	I felt like a stranger in my own body
I	53	YES	disease specific	Body Perception	Me senti como se qualquer coisa pudesse me afetar	I felt like anything could affect me
I	64	NO	new	Body Perception	Senti que não tenho saúde	I felt that I am not healthy
I	69	NO	new	Body Perception	Me senti fraco	I felt weak
I	43	NO	disease specific	Emotional	Me senti azedo	I felt sour
I	44	NO	disease specific	Emotional	Me senti zangado	I felt angry
I	49	NO	disease specific	Emotional	Chorei mais do que de costume	I cried more than usual
I	63	NO	new	Emotional	Me senti desequilibrado	I felt unbalanced
I	74	NO	new	Emotional	Me senti preso	I felt trapped
I	76	NO	new	Emotional	Me senti orgulhoso	I felt proud
I	78	YES	new	Emotional	Me senti com raiva	I felt angry
I	83	NO	new	Emotional	Me senti envergonhado	I felt ashamed
I	39	YES	disease specific	Fear and Powerlessness	Me senti fora de controle	I felt out of control
I	40	YES	disease specific	Fear and Powerlessness	Me senti com o corpo frágil	I felt my body fragile
I	48	YES	disease specific	Fear and Powerlessness	Me senti sem forças	I felt strengthless
I	50	NO	disease specific	Fear and Powerlessness	Me senti sem sorte	I felt unlucky
I	51	YES	disease specific	Fear and Powerlessness	Me senti vulnerável	I felt vulnerable
I	58	NO	disease specific	Fear and Powerlessness	Tive medo de fazer esforço físico	I was afraid of doing exercises
I	66	NO	new	Fear and Powerlessness	Me senti sem saber o que esperar	I didn’t know what to expect
I	73	NO	new	Fear and Powerlessness	Me senti com pavor	I felt terrified
I	77	NO	new	Fear and Powerlessness	Me senti apreensivo	I felt apprehensive
I	79	NO	new	Fear and Powerlessness	Me senti impotente	I felt helpless
I	92	YES	new	Fear and Powerlessness	Me senti assustado	I felt scared
I	31	YES	disease specific	Introvert	Me senti inseguro	I felt insecure
I	32	YES	disease specific	Introvert	Me senti com pena de mim mesmo	I felt sorry for myself
I	33	YES	disease specific	Introvert	Me senti em uma situação desesperadora	I felt in a desperate situation
I	34	YES	disease specific	Introvert	Fiquei com humor muito variável	I was in a very variable mood
I	54	YES	disease specific	Lifestyle	Mudei meus hábitos de atividade física	I changed my exercising habits
I	56	YES	disease specific	Lifestyle	Mudei meus hábitos alimentares	I changed my eating habits
I	72	YES	new	Negative impact on relations	Me senti sendo julgado	I felt that I was being judged
I	75	YES	new	Negative impact on relations	Me senti sendo forçado a fazer coisas que não quero	I felt being forced to do things I don’t want to do
I	84	YES	new	Negative impact on relations	Me senti controlado pelos outros	I felt that I was controlled by others
I	86	YES	new	Negative impact on relations	Me senti excluído	I felt excluded
I	88	NO	new	Neutral impact on relations	Me senti diferente	I felt different
I	41	NO	disease specific	Perception of age	Senti que a idade chegou	I felt that old age has come
I	47	NO	disease specific	Perception of age	Me senti mais velho do que sou	I felt older than I am
I	85	NO	new	Postitive impact on relations	Me senti apoiado	I felt supported
I	87	NO	new	Postitive impact on relations	Me senti cuidado	I felt being cared for
I	89	NO	new	Postitive impact on relations	Me senti importante	I felt important
I	65	NO	new	Results of diagnosis	Me senti em dúvida	I felt in doubt
I	80	NO	new	Results of diagnosis	Me senti surpreso	I felt surprised
I	1	YES	core	Sense of dejection	Me senti preocupado	I felt worried
I	10	YES	core	Sense of dejection	Me senti com a sensação de que o tempo não passava	I felt that time was not passing
I	12	YES	core	Sense of dejection	Me senti triste	I felt sad
I	19	YES	core	Sense of dejection	Me senti sem iniciativa	I felt without initiative
I	20	NO	core	Sense of dejection	Me senti sem vontade	I felt unwilling
I	21	NO	core	Sense of dejection	Me senti deprimido	I felt depressed
I	62	YES	new	Sense of dejection	Me senti culpado	I felt guilty
I	67	YES	new	Sense of dejection	Me senti desmotivado	I felt unmotivated
I	68	YES	new	Sense of dejection	Me senti desestimulado	I felt discouraged
I	70	NO	new	Sense of dejection	Me senti frustrado	I felt frustrated
I	71	YES	new	Sense of dejection	Me senti indiferente	I felt indifferent
I	82	YES	new	Sense of dejection	Me senti chateado	I felt upset
I	91	NO	new	Sense of dejection	Me senti culpado por não cuidar de mim mesmo como deveria	I felt guilty for not taking care of myself as I should
I	94	YES	core	Sense of dejection	Me senti incomodado	I felt bothered
I	27	YES	core	Sexual	Tive menos desejo sexual	I had less sexual desire
I	59	YES	disease specific	Sexual	Me senti insatisfeito com minha vida sexual	I felt dissatisfied with my sex life
I	8	YES	core	Single Items	Fuji dos meus pensamentos me ocupando com tarefas práticas do dia-a-dia	I ran away from my thoughts, busy with day-to-day practical tasks
I	28	YES	core	Single Items	Dias faltados no trabalho	Days missed at work
I	35	YES	disease specific	Single Items	Me senti mais cansado do que de costume	I felt more tired than usual
I	36	YES	disease specific	Single Items	Guardei meus pensamentos só pra mim	I kept my thoughts just for myself
I	45	YES	disease specific	Single Items	Me senti como se estivesse no vazio	I felt like I was in the void
I	52	YES	disease specific	Single Items	Me senti fragilizado	I felt weak
I	55	YES	disease specific	Single Items	Pensei na morte	I thought about death
I	60	YES	new	Single Items	Pensei na minha fé	I thought of my faith
I	81	YES	new	Single Items	Me senti tranquilo	I felt calm
I	95	YES	new	Single Items	Você tem pressão alta?	Do you have a high blood pressure?
I	7	NO	core	Sleep	Dormi mal à noite	I slept badly at night
I	17	NO	core	Sleep	Tive dificuldade de pegar no sono	I had difficulty falling asleep
I	23	NO	core	Sleep	Acordei cedo demais	I woke up too early
I	26	NO	core	Sleep	Passei a maior parte do tempo acordado	I spent most of the time awake
**Part**	**Item number**	**Included in final version**	**Questionnaire of origin**	**Domain**	**Brazilian Portuguese version**	**English ad hoc translation**
II	108	YES	disease specific	Empathy	meu sentimento de responsabilidade pela minha família ficou	my sense of responsibility for my family became …
II	111	YES	disease specific	Empathy	minha compreensão dos problemas alheios ficou	my understanding of other people’s problems became …
II	113	YES	disease specific	Empathy	a minha capacidade de ouvir problemas alheios ficou	my ability to hear other people’s problems became …
II	96	YES	core	Existential values	eu fiquei pensando na vida	I kept thinking about life...
II	97	YES	core	Existential values	minha alegria de viver ficou	my joy of living became …
II	103	YES	core	Existential values	a minha visão do futuro ficou	my vision of the future became …
II	104	YES	core	Existential values	a minha sensação de bem-estar ficou	my sense of well-being became …
II	105	YES	core	Existential values	a minha percepção sobre a vida ficou	my perception of life became …
II	106	YES	core	Existential values	o valor que dou a vida ficou	the value I give in life became …
II	125	YES	new	Existential values	me sinto como se não fosse mais normal	I feel like I’m not normal anymore...
II	126	YES	new	Existential values	me sinto como se não fosse mais o mesmo	I feel like I’m not the same anymore...
II	132	NO	new	Hypertension related	minha ansiedade com relação a pressão alta ficou	my anxiety about high blood pressure got...
II	133	NO	new	Hypertension related	penso que eu não tenho pressão alta	I think I don’t have high blood pressure...
II	107	YES	disease specific	Impulsive	a minha energia ficou	my energy became …
II	109	YES	disease specific	Impulsive	tenho aproveitado a vida	I have enjoyed life...
II	112	YES	disease specific	Impulsive	me sinto impulsivo	I feel impulsive...
II	114	YES	disease specific	Impulsive	a minha vontade de me envolver com algo novo ficou	my desire to get involved with something new became …
II	115	YES	disease specific	Impulsive	a minha vontade de me envolver com algo arriscado ficou	my desire to get involved with something risky got …
II	116	YES	disease specific	Impulsive	tenho feito coisas que utrapassam meus limites	I’ve been doing things that push my limits...
II	117	YES	new	Patient Role	frequento consultas médicas	I go to doctor’s appointments...
II	118	YES	new	Patient Role	faço exames	I do laboratory tests...
II	119	NO	new	Patient Role	me sinto fazendo mal para mim mesmo	I feel bad for myself...
II	120	YES	new	Patient Role	me sinto com dificuldades em seguir orientações médicas	I have difficulty following medical advices...
II	121	YES	new	Patient Role	me sinto cuidando de mim mesmo	I feel taking care of myself...
II	122	YES	new	Patient Role	tomo medicamentos	I take medicines...
II	123	YES	new	Patient Role	me sinto dependente de remédios	I feel dependent on medicines...
II	124	YES	new	Patient Role	me sinto confiante em orientações médicas	I feel confident in medical advice...
II	99	YES	core	Personal Relations	a minha relação com a minha família ficou	my relationship with my family became …
II	100	YES	core	Personal Relations	a minha relação com meus amigos ficou	my relationship with my friends became …
II	101	YES	core	Personal Relations	a minha relação com outras pessoas ficou	my relationship with other people became …
II	127	YES	new	Preoccupation with health	me sinto preocupado com sintomas de pressão alta	I feel worried about symptoms of high blood pressure...
II	128	YES	new	Preoccupation with health	me sinto preocupado com meus hábitos e estilo de vida	I feel worried about my habits and lifestyle...
II	129	YES	new	Preoccupation with health	me sinto preocupado com os tratamentos	I feel worried about the treatments...
II	98	NO	core	Relaxed/Calm	me senti tranquilo	I felt tranquil...
II	102	NO	core	Relaxed/Calm	me senti calmo	I felt calm...
II	110	NO	core	Relaxed/Calm	me sinto aliviado	I feel relieved...
II	130	YES	new	Single Items	meu desempenho no trabalho ficou	my work performance became …
II	131	YES	new	Single Items	minha prática sexual ficou	my sexual practice became …

all items in the item pool in Brazilian Portuguese and the English *ad hoc* translations. Domains of each part are in alphabetical order

### Sample

In this study, our target was a sample of the Brazilian population, and the inclusion criteria were: to be older than 18 years old, to be healthy (no self-reported health condition) and to have only hypertension (self-reported hypertension and no other self-reported comorbidity). We collected information about age, gender, ethnic origin, self-reported presence of hypertension, comorbidities, time from diagnosis of hypertension and level of education. A draft questionnaire composed of all the items in the item pool was sent to a target population by using the following strategies. We first used the Survey Monkey® Internet-based questionnaire manager to format digital and printed versions of the questionnaire and then distributed it in different media platforms, such as e-mails, WhatsApp® messages and Facebook® invitations. All invitations included a link to the digital questionnaire and could be forwarded to other people. We targeted healthy people and people living with hypertension, but we accepted responses from everyone and used the collected information to separate our target population from the rest afterwards. We also distributed printed versions of the questionnaire among the community healthcare workers around four different primary healthcare clinics. All questionnaires were self-applied. Data were collected in 2017. The responses in the printed versions were transcribed to the data bank by the first author. The draft questionnaire included an informed consent form and sociodemographic items.

### Measures

We selected Rasch model analysis [29] to screen the items and to establish the psychometric properties of this questionnaire because given that it assumes unidimensionality (Rasch models assume that all items reflect an underlying construct), it allows to investigate the fit of the items to a hypothesised dimension and how these items are interrelated and ordered on a latent continuum; thus, it supports the addition of the raw scores of items into a single score [30].

We referred to the qualitative material whenever an item did not fit the model and tried to understand why they did not fit. We aimed at two features of the Rasch models during the psychometric analysis: local response dependence (LD) [31] and differential item functioning (DIF) [32]. LD occurs when two items capture unique common information independently from what is supposed to be measured by the item set. That is, the answer of an item should not influence the answer of another item. Meanwhile, DIF occurs when the expected responses of individuals with the same level (but belong to different groups defined by an external factor) for a measured construct differ. That is, an external factor should not influence the answer of an item [33]. We included age (defined as age above or below 40), gender (male or female), ethnicity and the presence or absence of hypertension in our analysis.

To provide the measurement of psychosocial consequences consistent with Rasch measurement theory, the subscales calculated from the data collected for psychometric analysis should fit a graphical Rasch model (GRM) [34–36]. The overall model fit was assessed using the Andersen conditional likelihood ratio test [37] and the individual item fit was evaluated by comparing observed and expected item-rest score associations [22].

We also evaluated item fit graphically by dividing the sample into five score groups. For each item, we plotted the item mean score in each interval and compared all the scores to 95% confidence regions of the model expectations. For each item, the observed mean score in each class interval was plotted as a line together with a shaded area that indicates the 95% confidence region of the model expectations. Thus, when curves are contained in the shaded area, the observed data match the model expectations and thus indicate item fit.

The following was the modelling strategy:

(i) evaluating the fit of the COS core items in their previously identified domains to the Rasch models;
(ii) evaluating the fit of the COS core items to a GRM derived using item screening procedure, assessing the issues of COS core problematic items and removing them from the subscale;
(iii) adding COS disease-specific items to the subscale;
(iv) evaluating the fit of the COS disease-specific (+ COS core) items to the GRM, assessing the issues of COS disease-specific problematic items and removing them from the subscale;
(v) adding new items to the subscale;
(vi) evaluating the fit of the new items (new + COS items) to GRM, assessing the issues of problematic items and removing them from the subscale;
(vii) if possible, confirming the dimensionality of the derived subscales by using CFA;
(viii) evaluating reliability using Cronbach’s coefficient alpha.

After the Rasch model analysis, we used in each subscale CFA to confirm the fit indices and Cronbach’s alpha to test reliability.. In CFA and Cronbach’s alpha, missing data were excluded, and only complete responses were assessed. We used the evidence of local dependence found in the Rasch model analysis to indicate the correlated error terms in the CFA model. CFA was used only for subscales with four or more items after the Rasch model analysis. Rasch model analysis was conducted using the computer programme DIGRAM [38]. CFA and Cronbach’s alpha were conducted in STATA.

The null hypothesis of the statistical tests in the Rasch model analysis was that the model fits. We adjusted *p*-values by using the Benjamini-Hochberg [39] procedure to control the false discovery rate at 5% and thus took values above 0.05 as cut-off values for model fit. In CFA, the cut-off values were 0.06 for RMSEA and 0.95 for CFI [40]. Values above 0.70 for Cronbach’s coefficient alpha were considered adequate [41].

## Results

### Sample

We collected 1118 responses. After the exclusion of 319 informants with comorbidities, the validation sample consisted of 798 respondents living in all five Brazilian regions and 26 states that were recruited via different media platforms in the following proportion: 47.1% responded via the WhatsApp® link, 36.7% responded via the Facebook® link, 9.7% responded to the email invitation and 6,4% responded to the paper version.

Out of the 798 respondents, 285 (35.7%) were diagnosed with hypertension, 597 (74.8%) were female, 460 (57.6%) were over 40 years old, 566 (70.9%) were Caucasian and 204 (25.5%) had less than 11 years of education. All 798 respondents completed Part I, whereas 285 respondents with hypertension completed Part II. (Table 2. Population characteristics).

**Table 2:** Population characteristics

Characteristics	no hypertension *n* = 513		hypertension *n* = 285	
mean age, years	39.4 (18–73)		53.0 (20–85)	
mean education, years	17.6 (0–32)		11.7 (0–30)	
mean time from diagnosis, years	–		10.1 (0.1–40)	
Gender				
male	138	27%	63	22%
female	375	73%	222	78%
Ethnic origin				
afro + multi	124	24%	104	36%
caucaso + asian	386	75%	180	63%
Response media				
e-mail	67	13%	11	4%
Facebook	140	27%	153	54%
printed	3	1%	48	17%
WhatsApp	303	59%	73	26%

mean age, education, and time from diagnosis. Frequency of gender, ethnicity, and response media in the tow groups: ‘no hypertension’ and ‘hypertension’

**Fig. 1:**
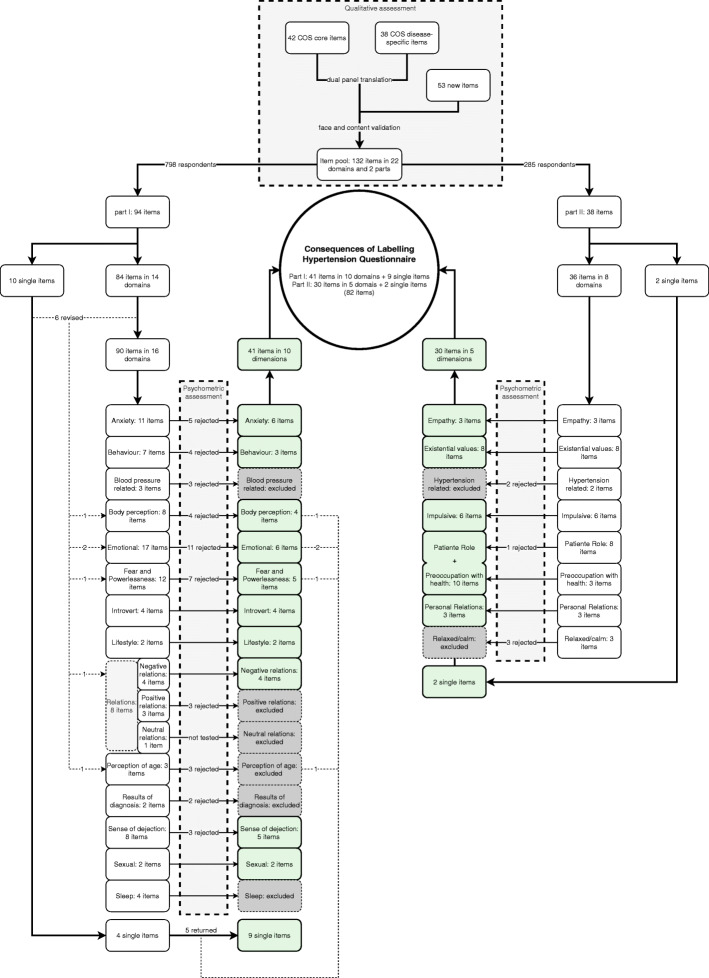
describes all the processes of the development of the questionnaire

Forty-four (46.8%) of the 94 items in Part I were rejected; thus, 41 items in 10 dimensions (Fig. 1): ‘anxiety’, ‘behaviour’, ‘body perception’, ‘emotional’, ‘fear and powerlessness’, ‘introvert’, ‘lifestyle’, ‘negative relations’, ‘sense of dejection’ and ‘sexual’ and 9 single items remained.

Six (15.7%) of the 38 items in Part II were rejected; thus 30 items in five dimensions (Fig. 1): ‘empathy’, ‘existential values’, ‘impulsive’, ‘patient role + preoccupation with health’ and ‘personal relations’ and two single items remained. A 71-item questionnaire with two parts was yielded, with 15 dimensions and 11 single items. The main reason for the exclusion of items was 65% of the cases failed to fit, followed by 30% of DIF cases. All DIF cases were found in the items of Part I. The main variable responsible for DIF was the presence of hypertension found in seven of the 17 items that were excluded for this reason. Age was responsible for DIF in five items, gender in three and ethnicity in two items. (Table 3. Rejected items and reasons for the exclusion).

**Table 3:** Rejected items and reasons for the exclusion

Part	Domain	Q of origin	item number	reason for exclusion
I	Anxiety	core	2	no fit
		core	3	no fit
		core	4	no fit
		core	93	too many missing responses
		new	61	no fit
	Behaviour	core	6	DIF
		core	9	DIF
		core	11	DIF
		core	18	DIF
	Blood pressure related	new	30	DIF
		new	57	DIF
		new	90	DIF
	Body Perception	disease specific	35	DIF
		disease specific	42	DIF
		new	64	DIF
		new	69	no fit
	Emotional	disease specific	43	no fit
		disease specific	44	no fit
		disease specific	49	DIF
		new	63	no fit
		new	74	no fit
		new	76	no fit
		new	83	DIF
		new	70	no fit
		new	81	no fit
		disease specific	36	no fit
		disease specific	52	no fit
	Fear and Powerlessness	disease specific	50	DIF
		disease specific	58	DIF
		new	66	no fit
		new	73	no fit
		new	77	no fit
		new	79	no fit
		disease specific	52	no fit
	Introvert	disease specific	36	DIF
	Perception of age	disease specific	41	no fit
		disease specific	47	no fit
		disease specific	45	no fit
	Positive relations	new	85	no fit
		new	87	no fit
		new	89	no fit
	Results of diagnosis	new	65	no fit
		new	80	no fit
	Sense of dejection	core	20	19 fits better than 20
		core	21	DIF
		new	91	DIF
	Sleep	core	7	no fit
		core	17	no fit
		core	23	no fit
		core	26	no fit
	Social Relations	new	88	neutral
II	Hypertension related	new	132	no fit
		new	133	no fit
	Patient Role	new	119	no fit
	Relaxed/Calm	core	98	no fit
		core	102	no fit
		core	110	no fit

The graphical model check showed that as the domain score increased, items’ mean scores also increased, indicating that all items within a domain measure the same construct. All plots are presented in the Additional file 1.

### Measures

#### Part I

##### Rasch model analysis

We had 10 single items for Part I that were derived from the content validation study. Based on the qualitative assessment of the item pool, we hypothesised that six of them (35, 36, 45, 52, 75 and 81) could be tested in the following domains—35 in ‘body perception’, 36 in ‘emotional’ and in ‘introvert’, 81 in ‘emotional’, 52 in ‘emotional’ and in ‘fear and powerlessness’, 45 in ‘perception of age’ and 75 in ‘negative relations.’ Item 75 found fit in the domain, whereas the five other items were rejected in the tested domains. The nine items that failed to find a place in a subscale were kept as single items.

Twenty-eight items derived from COS core were tested in five different dimensions: ‘anxiety’, ‘behaviour’, ‘sense of dejection’, ‘sexual’ and ‘sleep.’ The ‘sleep’ dimension, which was composed only by core items, did not fit the Rasch model analysis no matter the combination of the four sleep items. Fourteen of the remaining 24 items fitted the four other respective dimensions.

Twenty-eight items derived from other COS disease-specific questionnaires were tested in eight different dimensions: ‘anxiety’, ‘body perception’, ‘emotional’, ‘fear and powerlessness’, ‘introvert’, ‘lifestyle’, ‘perception of age’ and ‘sexual.’ Four of these dimensions had altogether 12 items rejected: ‘body perception’, ‘emotional’, ‘fear and powerlessness’ and ‘perception of age’, whereas 16 items fitted the respective domains.

Thirty-four new items were tested in nine different dimensions. Four of these were newly created dimensions: ‘blood pressure related’, ‘relations negative’, ‘relations positive’ and ‘results of the diagnosis.’ The five other dimensions that had new items tested were ‘anxiety’, ‘body perception’, ‘emotional’, ‘fear and powerlessness’ and ‘sense of dejection.’ Twenty-three new items were rejected, and 11 were accepted in the tested dimensions.

Eight items comprised the ‘social relations’ dimension (72, 75, 84, 85, 86, 87, 88 and 89). The first analysis that included all items suggested two subscales with opposite relational effects and one neutral item. We then excluded the neutral item (88) and split the items in two dimensions: ‘positive relations’ with Items 85, 87 and 89 and ‘negative relations’ with the remaining Items 72, 75, 84 and 86. The ‘positive relations’ dimension failed to find fit, but the ‘negative relations’ found fit with DIF with age for Item 72 (being judged): those over 40 years old consistently scored lower than those under 40 who have the same total score.

Items 27 and 59 in the ‘sexual’ dimension showed DIF with gender. Women consistently scored higher on Item 27 and lower on Item 59 compared with men.

In the ‘emotional’ dimension, item pairs 67/68 and 78/82 had LD. In the ‘anxiety’ dimension, item pairs 25/29 also had LD. The same was revealed for item pair 22/24 in ‘behaviour’, item pairs 37/38 and 46/53 in ‘body perception’, item pair 75/84 in ‘negative relations’ and finally item pairs 10/19 and 12/19 in ‘sense of dejection.’ In all these cases, these pairs fitted the subscales.

Items 3 and 4 were different versions of the same item, and we included only one of them at a time in the ‘anxiety’ dimension. We began with two versions of the subscale, each with either Item 3 or 4 and then tried to add new items. However, in both versions, these items misfit and were excluded from the final version of the subscale. Items 19 and 20 were also two different versions of the original item. The ‘sense of dejection’ dimension showed good fit with Item 19.

The following domains had no items selected and were excluded from the final questionnaire: ‘blood pressure related’ with three items, ‘perception of age’ with two items, ‘positive relations’ with three items, ‘results of the diagnosis’ with two items, ‘sleep’ with four items and ‘neutral relations’ with one item. The results of the Rasch model analysis are shown in Table 4 with the selected set of items for each subscale.

**Table 4:** Selected items: part I

		Rasch model analysis results					Confirmatory Factor Analysis results		Internal consistency
	Scale	number of items tested	number of items selected	selected item’s number	CML	p	rmsea	CFI	Cronbach’s-alfa
**Part I**	Anxiety	11	6	13	32.2	0.046	0.062	0.986	0.839
				14					
				15					
				16					
				25					
				29					
	Behaviour	7	3	5	18.9	0.332			0.708
				22					
				24					
	Body perception	8	4	37	14.7	0.987	0,000	1000	0.806
				38					
				46					
				53					
	Emotional	17	6	62	54.2	0.015	0.049	0.987	0.851
				67					
				68					
				71					
				78					
				82					
	Fear and Powerlessness	12	5	39	34.2	0.063	0.090	0.978	0.837
				40					
				48					
				51					
				92					
	Introvert	5	4	31	17.1	0.106	0.047	0.997	0.804
				32					
				33					
				34					
	Lifestyle	2	2	54	5	0.418			0.599
				56					
	Negative relations	4	4	72	10.2	0.513	0,000	1000	0.777
				75					
				84					
				86					
	Sense of dejection	8	5	1	39.4	0.172	0.054	0.988	0.682
				10					
				12					
				19					
				94					
	Sexual	2	2	27	11.3	0.045			0.722
				59					

results of Rasch analysis, confirmatory factor analysis and internal consistency for the selected items in part 1. Refer to Table 1 for the items’ contents

##### CFA and reliability

Table 4 presents the CFA parameters for Part I. Two subscales had an RMSEA above 0.06 (‘anxiety’ and ‘fear and powerlessness’), whereas none had CFI below 0.95.

All 10 accepted subscales were tested for internal consistency with Cronbach’s alpha coefficients. Two subscales, ‘lifestyle’ and ‘sense of dejection’, had alpha values below 0.7.

#### Part II

##### Rasch model analysis

Twelve items derived from COS core were tested in three different dimensions: ‘existential values’, ‘personal relations’ and ‘relaxed/calm.’ The ‘relaxed/calm’ items neither fitted the Rasch model analysis nor formed a scale. All other core items found fit. Local dependence was observed between Items 103 and 104.

Nine items derived from COS disease-specific items were tested in two different dimensions: ‘empathy’ and ‘impulsive’. All items were accepted. Local dependence was found between Items 111 and 113.

Fifteen new items were tested in four different dimensions: ‘existential values’, ‘hypertension related’, ‘patient role’ and ‘preoccupation with health.’ The ‘existential values’ dimension was the only one that had items from more than one origin tested (core and new). Three of the items were rejected: one in the ‘patient role’ dimension (Item 119) and two in the ‘hypertension related’ dimension (Items 132 and 133). We had two single items for Part II, both new items.

The qualitative assessment of the items of ‘patient role’ and ‘preoccupation with health’ suggested that they could be all part of a combined scale called the ‘patient role + preoccupation with health’ subscale. The Rasch model analysis, where both scales were combined, had a nice fit with no DIF, resulting in a new 10-item subscale: Items 117, 118, 120, 121, 122, 123, 124, 127, 128 and 129.

The following domains had no items selected and were excluded from the final questionnaire: ‘hypertension related’ with two items and ‘relaxed/calm’ with three items.

##### CFA and reliability

Table 5 presents the CFA parameters for Part II. Two subscales had RMSEA above 0.06 (‘impulsive’ and ‘patient role + preoccupation with health’), whereas none had CFI below 0.95.

**Table 5:** Selected items: part II

		Rasch model analysis results					Confirmatory Factor Analysis results		Internal consistency
	Scale	number of items in the pool	number of items selected	selected item’s number	CML	p	rmsea	CFI	Cronbach-alfa
**Part II**	Empathy	3	3	108	6.6	0.712			0.733
				111					
				113					
	Existential values	8	8	96	31.0	0.124	0.054	0.982	0.860
				97					
				103					
				104					
				105					
				106					
				125					
				126					
	Impulsive	6	6	107	19.7	0.494	0.079	0.971	0.834
				109					
				112					
				114					
				115					
				116					
	Patient Role + Preoccupation with health	11	10	117	39.6	0.233	0.070	0.960	0.864
				118					
				120					
				121					
				122					
				123					
				124					
				127					
				128					
				129					
	Personal Relations	3	3	99	0.7	0.984			0.757
				100					
				101					

results of Rasch analysis, confirmatory factor analysis and internal consistency for the selected items in part 2. Refer to Table 1 for the items’ contents

All five accepted subscales were tested for internal consistency with Cronbach’s alpha coefficients described in Table 5. None had an alpha below 0.7.

## Discussion

### Major findings

A measurement tool, which covers psychosocial experiences after the diagnosis of hypertension, was developed and validated, encompassing a total of 82 items divided into two parts and 15 subscales (10 in Part 1 and five in Part 2). We established known-group validity for the total score and proved that the instrument discriminates well between cases and controls.

The final scale is a multidimensional group of subscales, which, in turn, are unidimensional. By dividing the multidimensional scale in unidimensional subscales, we identified the key elements of the psychosocial consequences (a multidimensional construct by definition) to provide content coverage and relevance. We also measured each element within their own unidimensional subscale.

This study revealed that being labelled with hypertension has common psychosocial consequences with having abnormal screening results for breast cancer, lung cancer, cervical cancer and aortic aneurism, all of which were previous targets of the four different COS versions [25, 27, 28, 42]. This finding is supported by the inclusion of COS ‘disease-specific’ items, which were accepted in the final version of the questionnaire. These results may also provide a comparison between the psychosocial consequences of labelling hypertension and the psychosocial consequences related to false positive results of screening related to such four conditions.

However, we do not expect that the new questionnaire, which is composed of new and inherited items from the COS family, is the same metric as the COS questionnaires. New items were generated, and they expanded the final version of the questionnaire, altering the composition of the item sets inherited from COS and thus measuring a different (but with similarities) construct from the COS versions. Hence, the psychosocial effects of labelling hypertension share similarities with the effects of being screened but are, to an extent, different from the other psychosocial effects measured by the COS questionnaires.

New subscales specifically relevant for people labelled with hypertension were developed. The subscale ‘relations negative’ strengthens the social aspects of the psychosocial consequences of labelling, whereas the subscale ‘patient role’ strengthens the labelling effects, suggesting that the labelled people develop actions and attitudes expected from the labelled condition. These relevant aspects are found in the qualitative content analysis of our previous study [12].

The scores generated from questionnaire scales are further valid if analyses based on item response theory (IRT) are conducted [30, 43, 44]. We used Rasch model analysis, one subgroup of IRT models. The selection of Rasch model analysis allowed us to start from our qualitatively developed domains, submit them in a survey and test if the response data fit the Rasch model [30].

All items were excluded using a data driven method. However, we found a strength, that is, our statistical psychometric analyses were not purely exploratory, but mostly confirmatory. We used Rasch model analysis to confirm our hypotheses: items were relevant, covered different aspects of the target outcome and worked well together. We referred to the qualitative material to analyse the impact of the exclusion on the subscales’ content coverage and to explore possibilities to fix the excluded items’ issues. Given that the developed subscales had adequate psychometric properties and enough items to allow for adequate content coverage, the excluded items may have their revised versions retested in the future.

The exclusion of items based on LD and DIF aim at including only items that are correlated through the latent trait, in this case, the psychosocial consequences of labelling hypertension composed of its identified sub-dimensions.

Traditionally, questionnaires are validated using analyses that are based on classical test theories, such as Cronbach’s alpha and CFA. These methods are insufficient to establish unidimensionality [45], but can be used complementarily to support the Rasch model analysis results. In this study, the derived subscales were confirmed using CFA but should ideally be confirmed in a new dataset. The overall result is that the CFA models confirmed the measurement models derived using Rasch model analysis. Internal consistency reliability was also confirmed for most of the subscales. However, two of them, ‘lifestyle’ and ‘sense of dejection’, had values of Cronbach’s coefficient alpha below 0.7, suggesting that they lack reliability. These subscales should be reviewed in the future. We also tested reliability with the sum of the scores of each sub-dimension for each of the questionnaire’s part with Cronbach’s alpha above 0.85, indicating that the subscales work well together.

The final set of items was composed of a long questionnaire, which might not be of practical use. If it proves to be a problem for future use, the 11 single items can be excluded because they also make the results difficult to interpret. Moreover, subscales that are composed of more than five items can be easily shortened to produce an easy-to-apply questionnaire. Long questionnaires may provide improved content validity and identify nuance in the psychosocial consequences of labelling hypertension. Future studies can aim to disclose floor/ceiling effects, supporting the qualitative evaluation of content coverage.

This study has certain limitations. Considering that the questionnaire was distributed online (mobile and personal computer), making clarifications whilst completing the items was difficult (although available) for the participants. In a scenario with a wide range of reading abilities, a self-applied questionnaire can be less accessible. Certain items also showed DIF with gender and age, indicating that when using this scale, we must be careful when comparing the effects between male and female and people with different ages. Item 5 was found to be wrongly translated during the analysis. Therefore, further tests are recommended for this item in the ‘emotional’ dimension. Another recommendation is to retest the ‘behaviour’ dimension without this item. Note that Item 14 in Part 2 was never tested.

Another limitation of this study is that the sampling was based on an open design because no control existed on whether the subjects had really undergone a diagnosis of hypertension; specifically, a diagnosis of mild hypertension. We intended to measure the impact of labelling and assumed that such an effect requires the subject to recognise himself or herself as hypertensive, and not that the correct diagnosis is clinically identified. This assumption is justifiable because previous literature and our own qualitative findings in previous steps of the development of this questionnaire suggested that the effect of hypertension labelling is unrelated to the correct diagnosis of hypertension [4]. Furthermore, the prevalence of mild hypertension among people without comorbidities is far greater than that of moderate and severe hypertension [46]. When we included only those without comorbidities, we expected to remove most people with moderate and severe hypertension.

## Conclusion

A new condition-specific questionnaire with a total of 82 items in 15 subscales was developed for people labelled with hypertension; the questionnaire had high content validity and adequate psychometric properties. This measure is called ‘Consequences of Labelling Hypertension Questionnaire’, which covers two parts of the psychosocial experiences after the diagnosis of hypertension. The adequate reliability, unidimensionality and invariant measurement of the subscales were demonstrated using Rasch model analysis. However, further examinations are required for the final subscales in a new dataset to confirm the results presented here and promote improvements to this questionnaire.

### Implications for clinical practice and research

This questionnaire is not designed to be used in clinical practice. However, research on the psychosocial consequences of labelling is relevant for clinical practice and for population studies. It is a tool that can be used in future research on hypertension, especially in scenarios of screening, preventive population strategies and in intervention studies that are willing to access all possible results of the interventions.

## Supplementary Information

**Additional file 1.** Plots: observed mean score in each class interval (line) and the 95% confidence region of the model expectations (shaded area).

## Data Availability

The datasets used and/or analyzed during the current study are available from the corresponding author on reasonable request.

## Abbreviations

CVD: Cardiovascular disease
COS: Consequences of screening
PROM: Patient reported outcome measure
IRT: Item response theory
CFA: Confirmatory factor analysis
LD: Local dependence
DIF: Differential item functioning
