# Supplementary material for: Psychometric properties of a condition-specific PROM for the psychosocial consequences of Labelling hypertension by using Rasch analysis
Source: J Patient Rep Outcomes. 2021 Feb 4;5:19. doi: 10.1186/s41687-021-00291-4 (PMC7862460; doi:10.1186/s41687-021-00291-4)
Supplement: Supplementary file 1 [file 41687_2021_291_MOESM1_ESM.rtf]

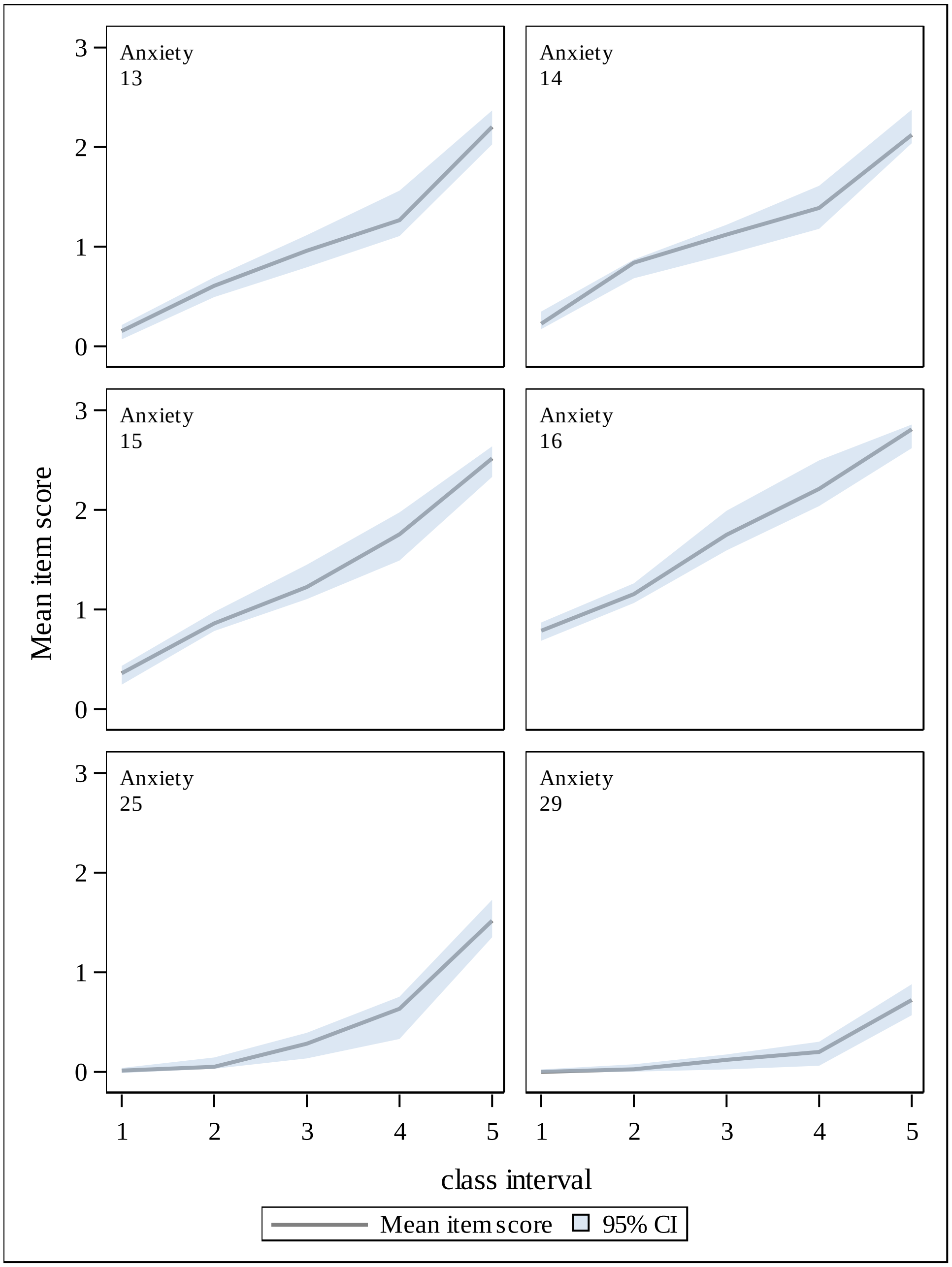
Anxiety 13
Anxiety 14
Anxiety 15
Anxiety 16
Anxiety 25
Anxiety 29
Mean item score
class interval
0
1
2
3
1
2
3
4
5
Mean item score
95% CI

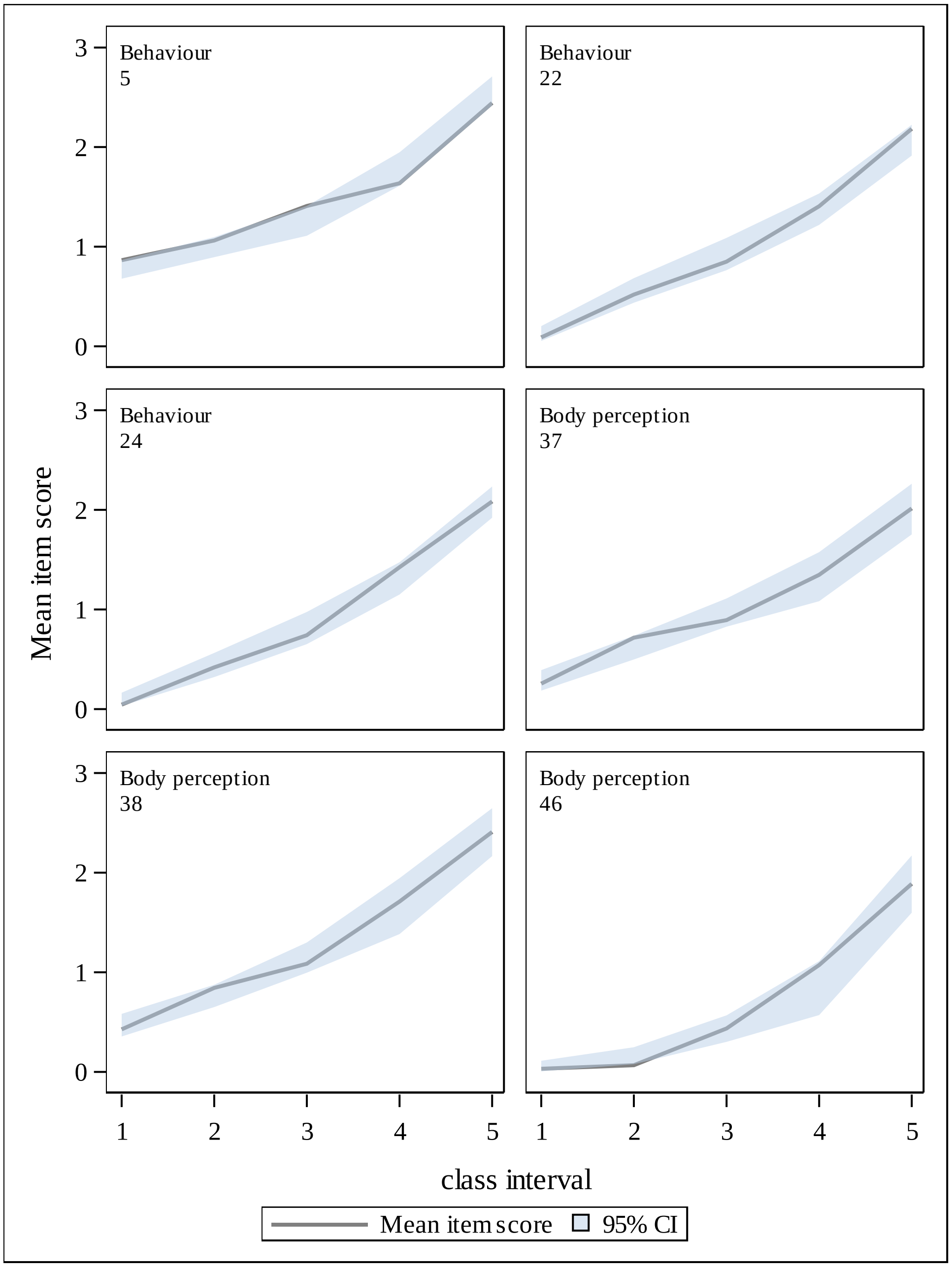
Behaviour
5
Behaviour
22
Behaviour
24
Body perception
37
Body perception
38
Body perception
46
Mean item score
0
1
2
3
1
2
3
4
5
class interval
Mean item score
95% CI

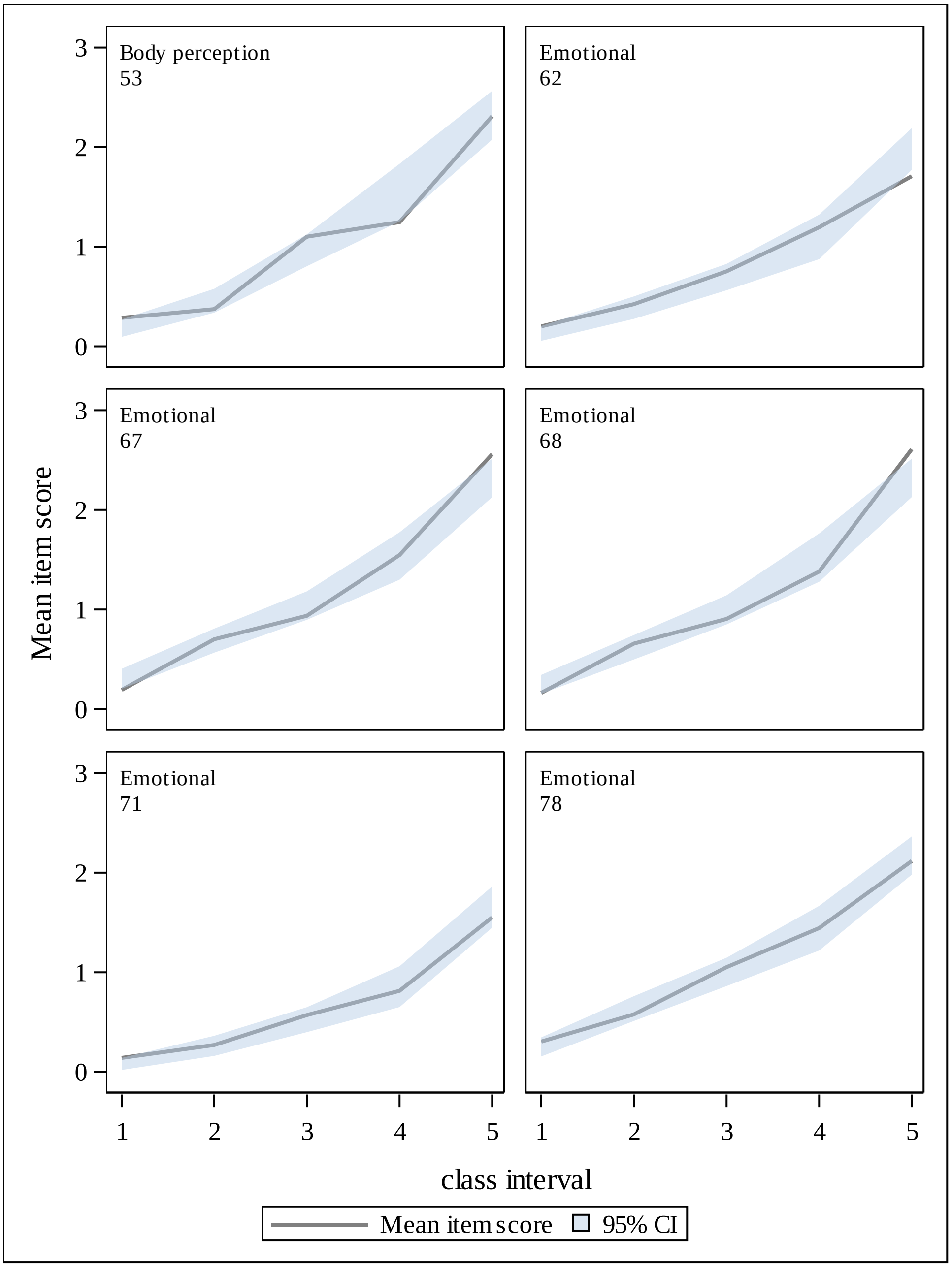
Body perception
53
Emotional
62
Emotional
67
Emotional
68
Emotional
71
Emotional
78
Mean item score
class interval
0
1
2
3
1
2
3
4
5
Mean item score
95% CI

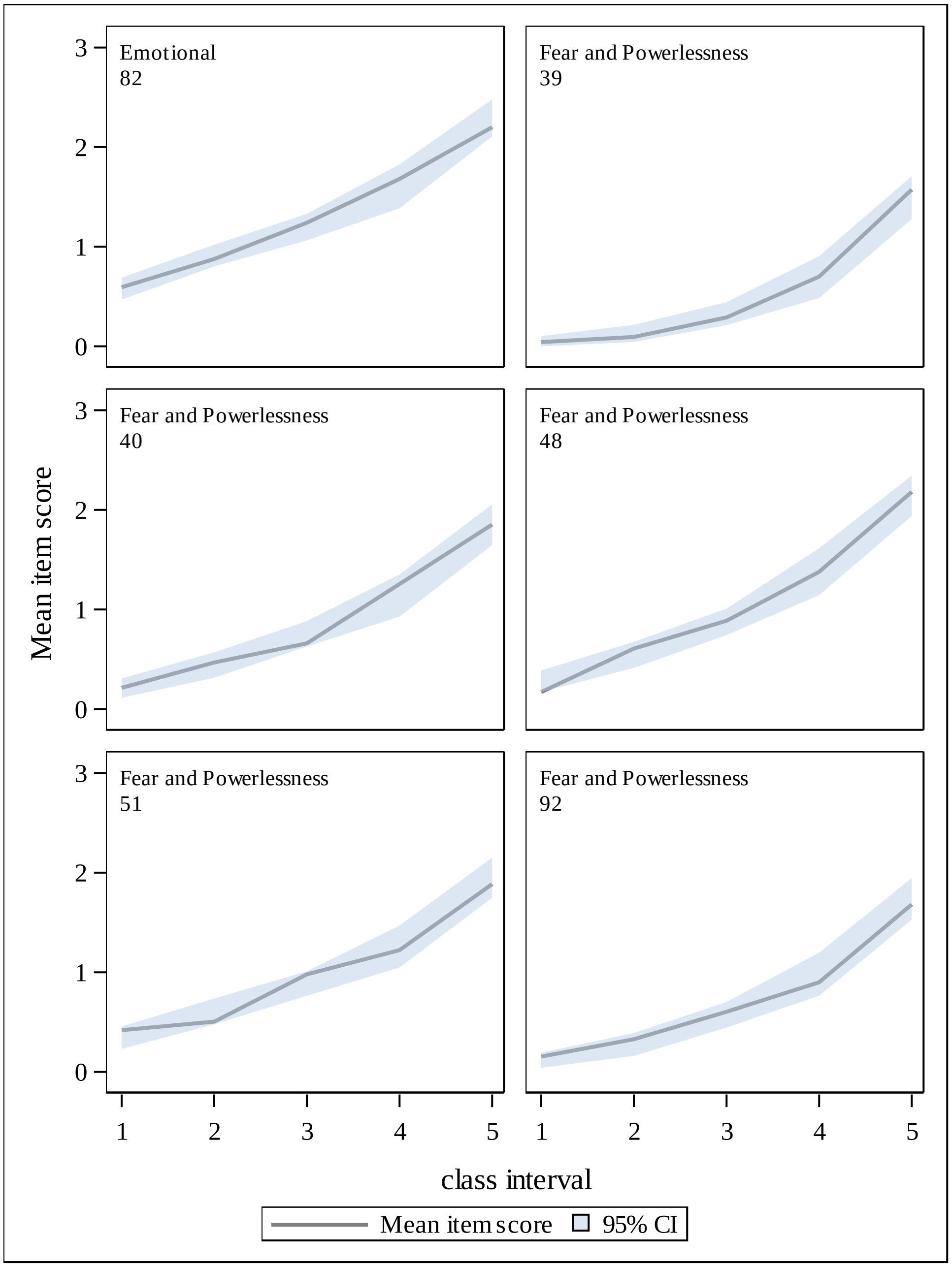
Emotional
82
Fear and Powerlessness
39
Fear and Powerlessness
40
Fear and Powerlessness
48
Fear and Powerlessness
51
Fear and Powerlessness
92
Mean item score
0
1
2
3
1
2
3
4
5
class interval
Mean item score
95% CI

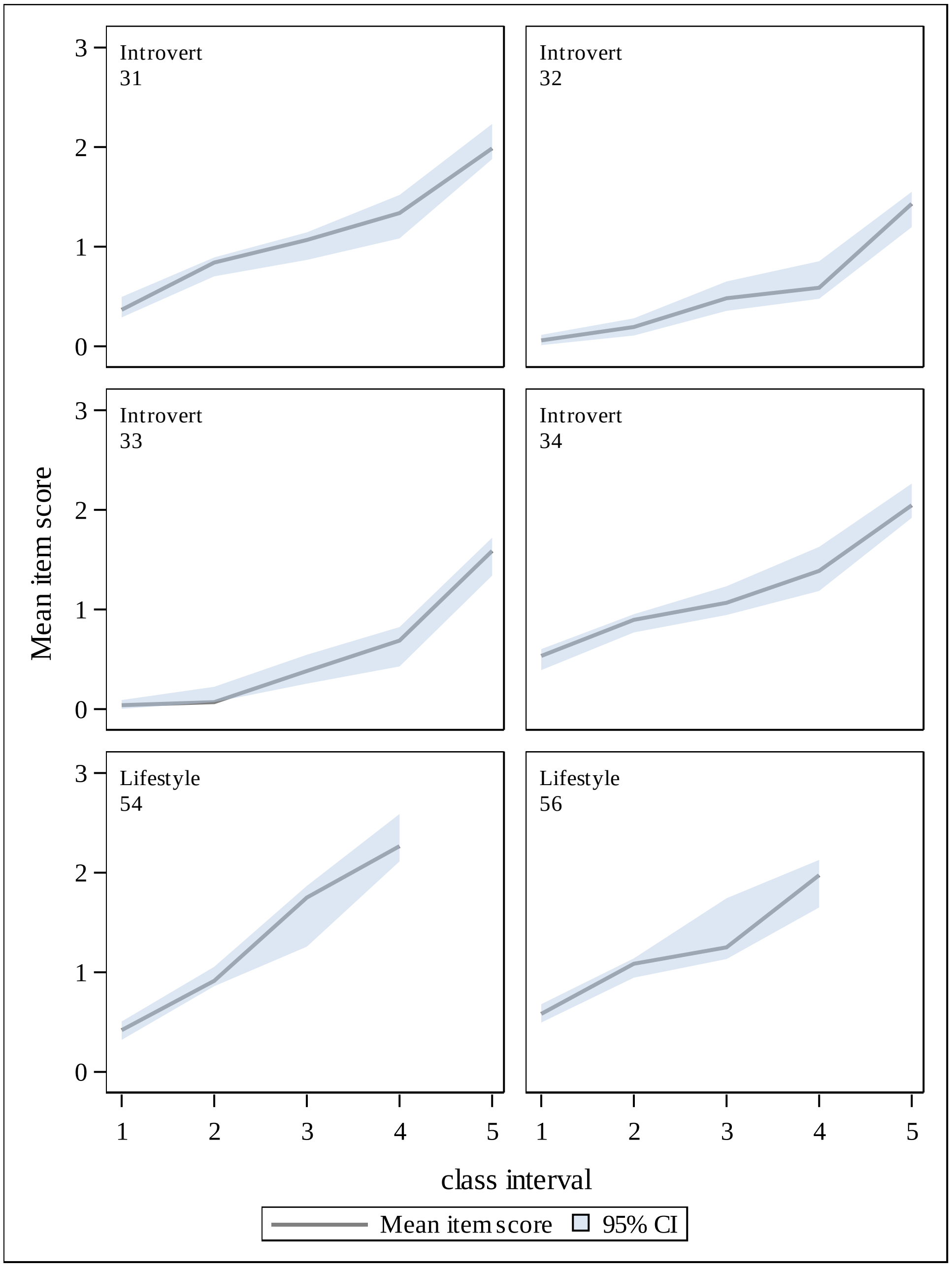
Introvert
31
Introvert
32
Introvert
33
Introvert
34
Lifestyle
54
Lifestyle
56
Mean item score
class interval
0
1
2
3
1
2
3
4
5
Mean item score
95% CI

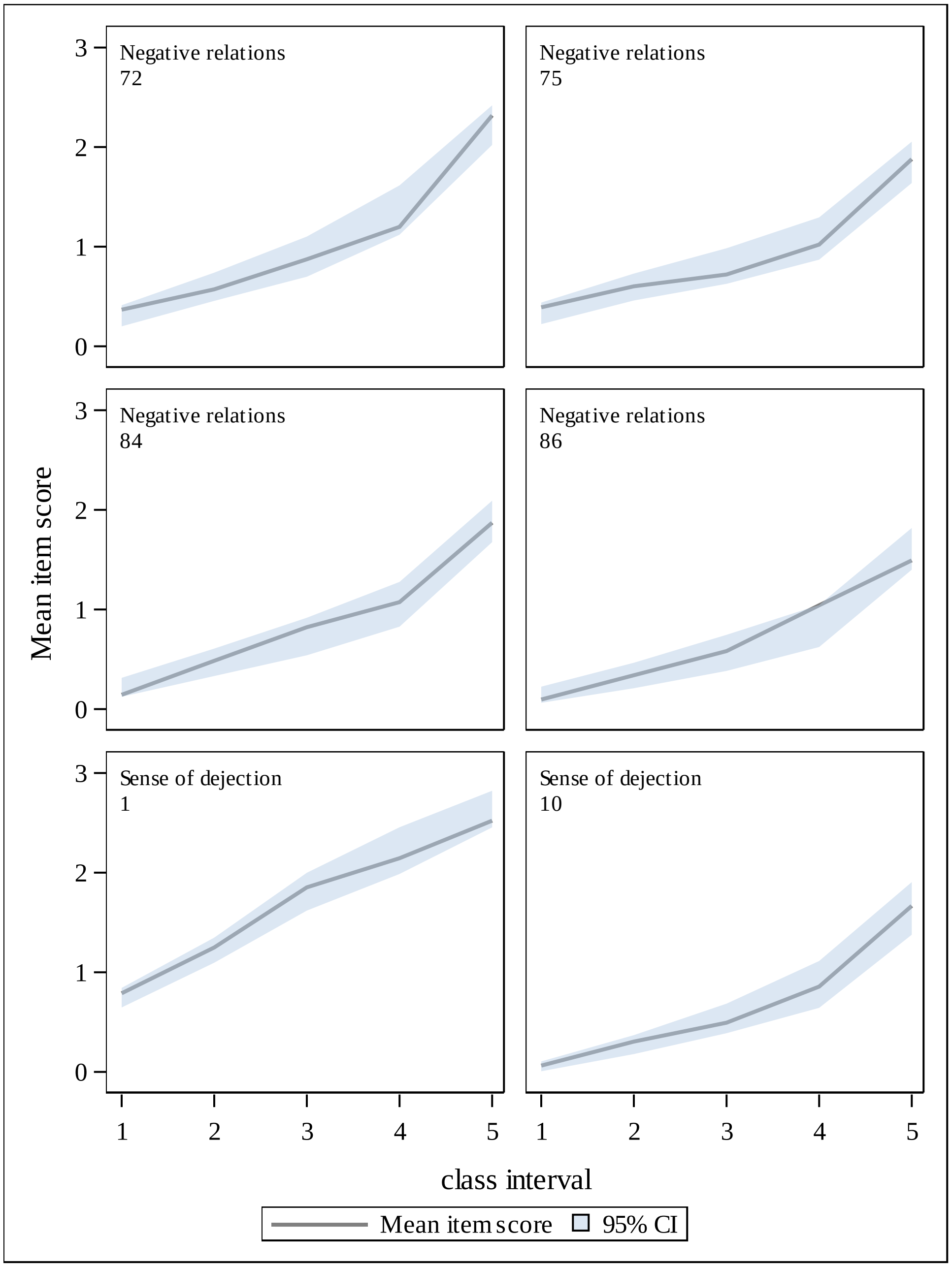
Negative relations
72
Negative relations
75
Negative relations
84
Negative relations
86
Sense of dejection
1
Sense of dejection
10
Mean item score
class interval
Mean item score
95% CI

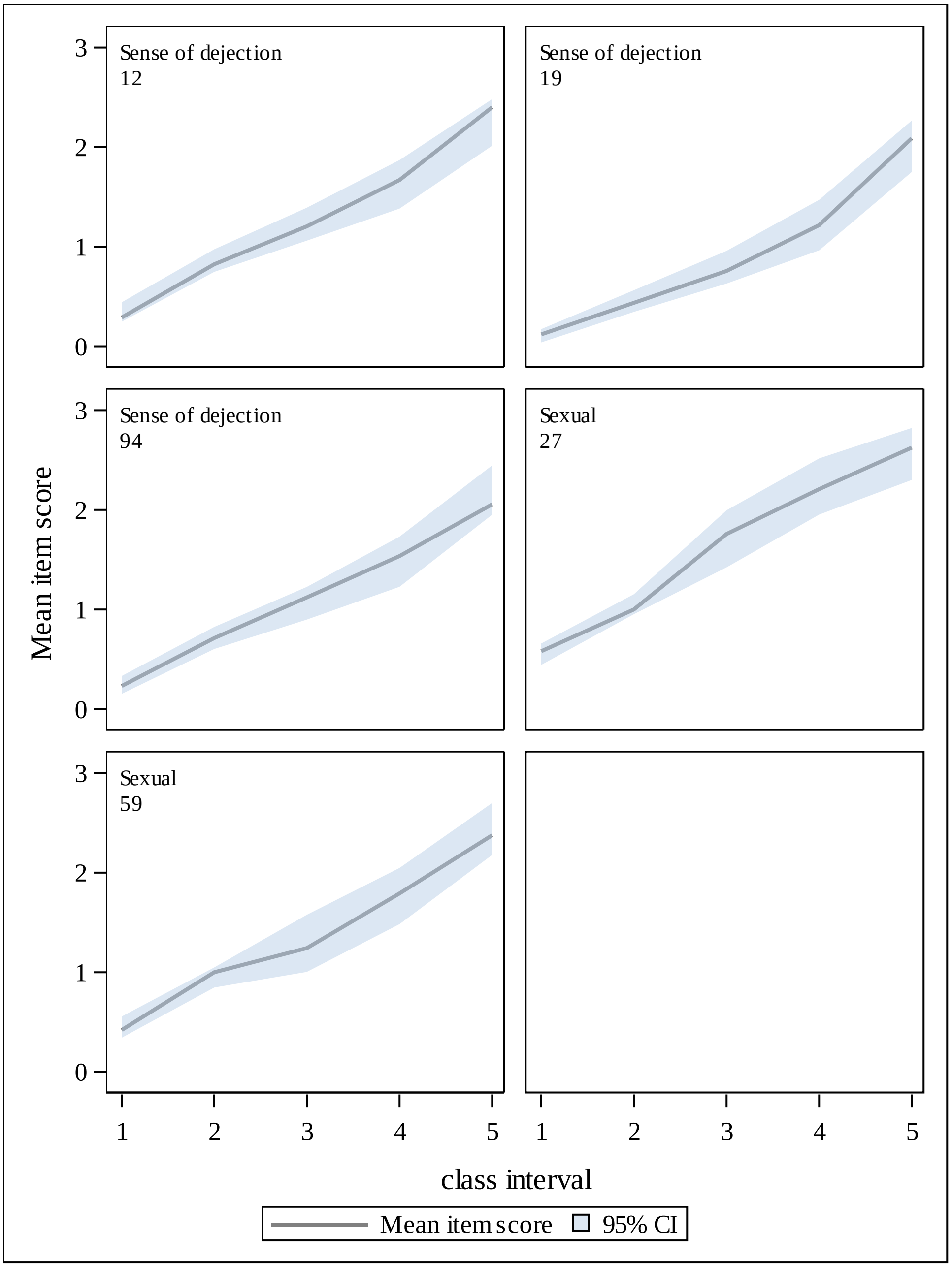
Sense of dejection
12
Sense of dejection
19
Sense of dejection
94
Sexual
27
Sexual
59
Mean item score
class interval
0
1
2
3
1
2
3
4
5
Mean item score
95% CI

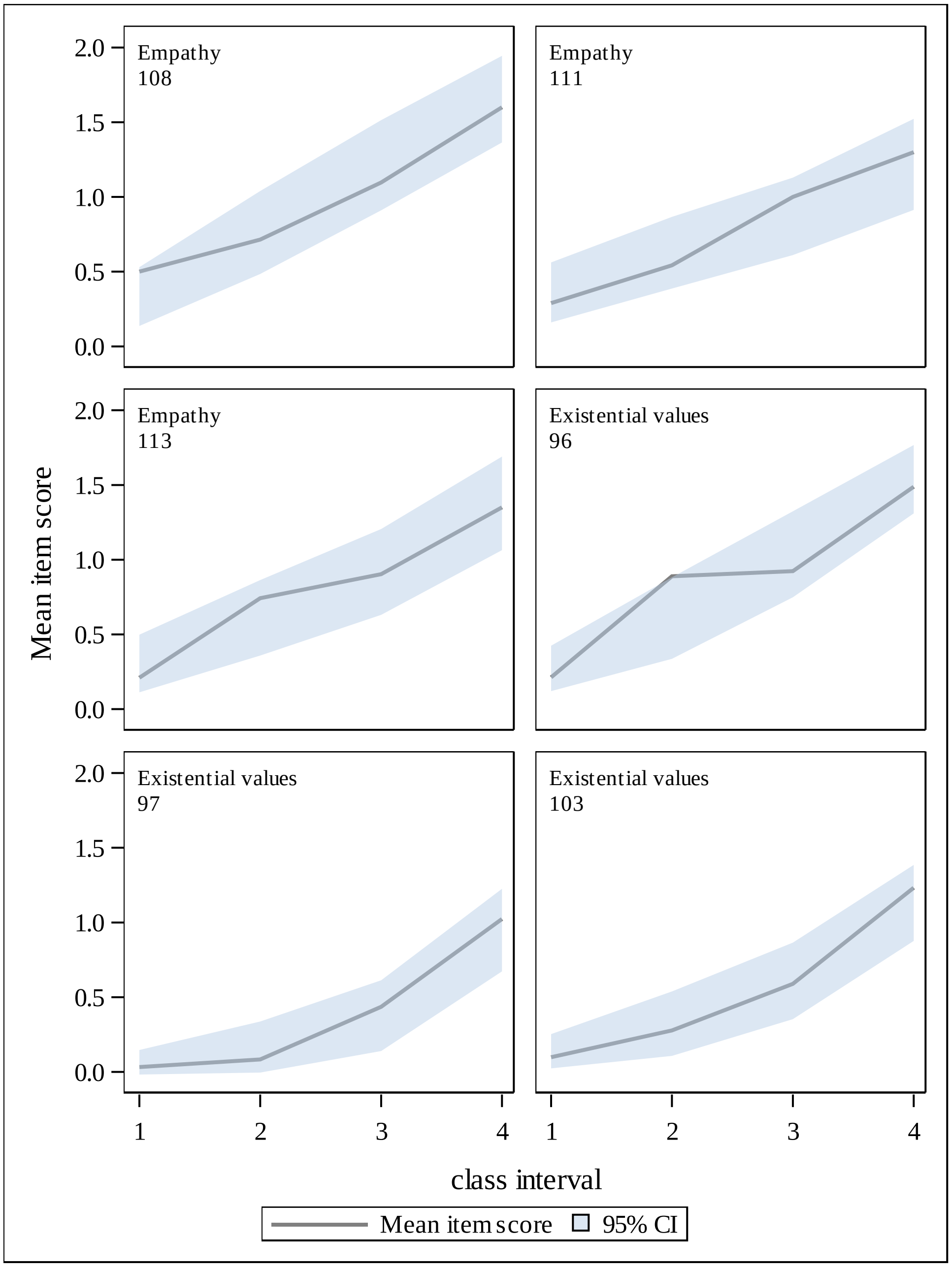

Empathy
108
Empathy
111
Empathy
113
Existential values
96
Existential values
97
Existential values
103
Mean item score
0.0
0.5
1.0
1.5
2.0
1
2
3
4
class interval
Mean item score
95% CI

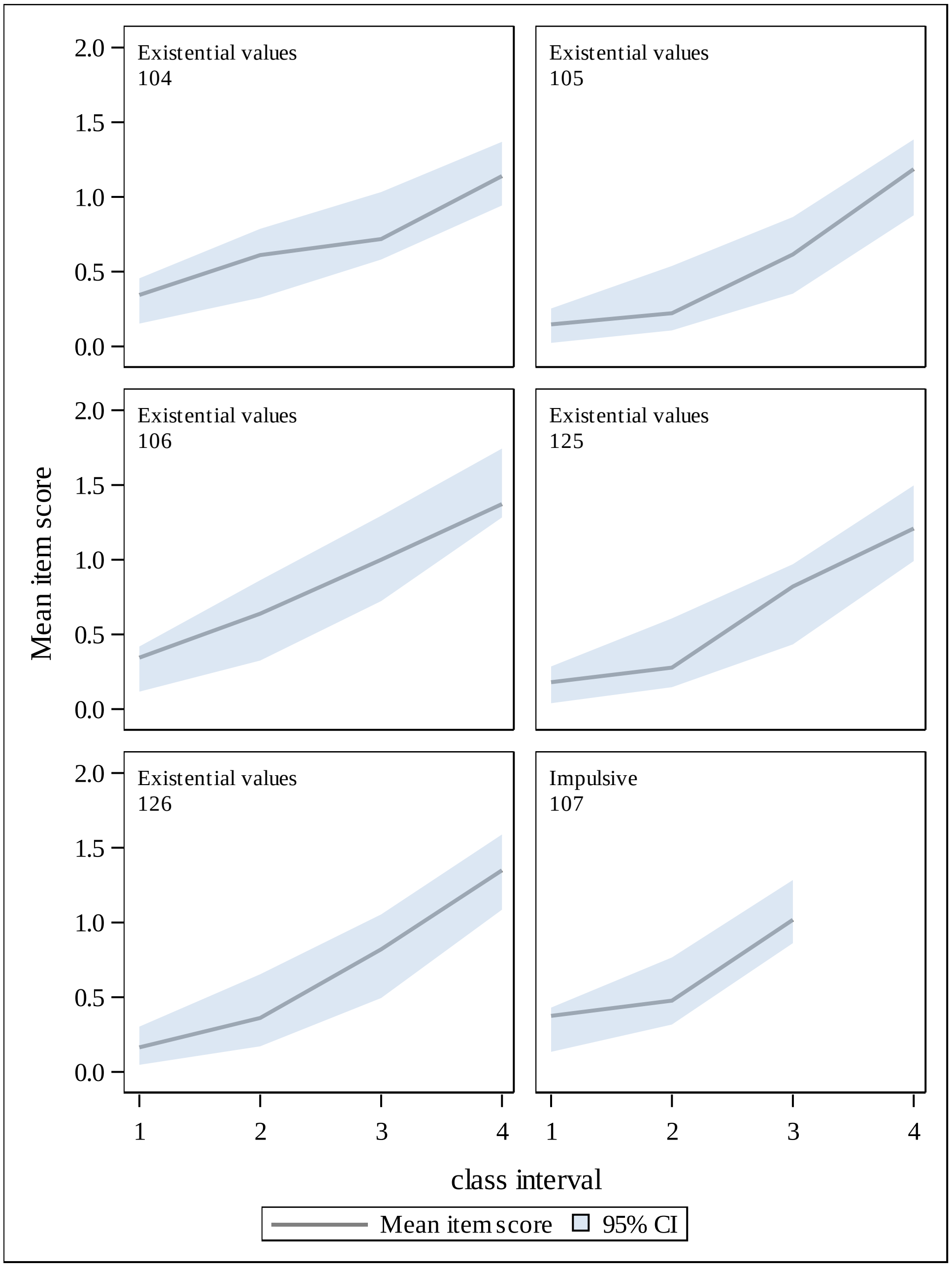
Existential values
104
Existential values
105
Existential values
106
Existential values
125
Existential values
126
Impulsive
107
Mean item score
2.0
1.5
1.0
0.5
0.0
1
2
3
4
class interval
Mean item score
95% CI

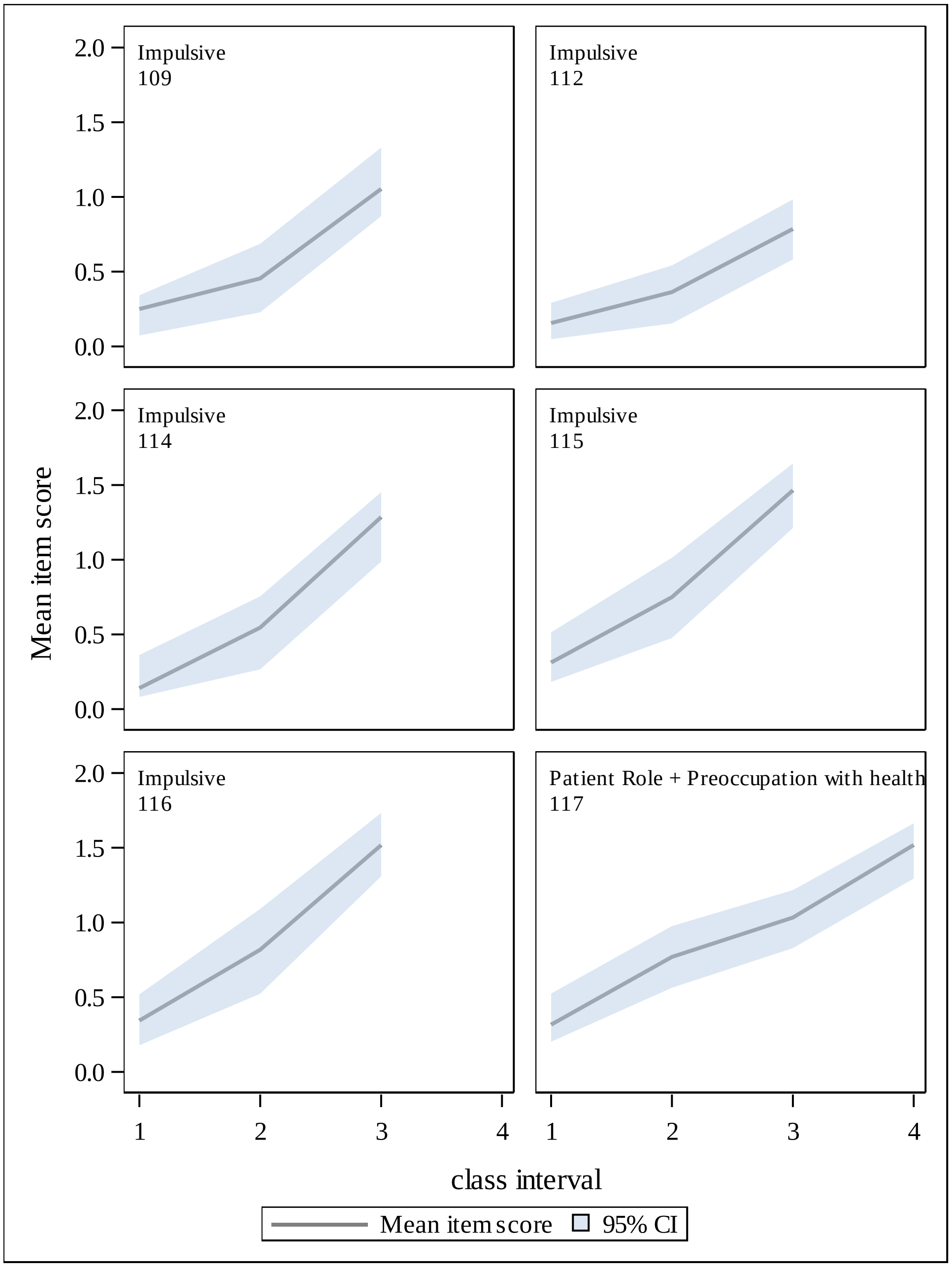
Impulsive
109
Impulsive
112
Impulsive
114
Impulsive
115
Impulsive
116
Patient Role + Preoccupation with health
117
Mean item score
2.0
1.5
1.0
0.5
0.0
1
2
3
4
class interval
Mean item score
95% CI

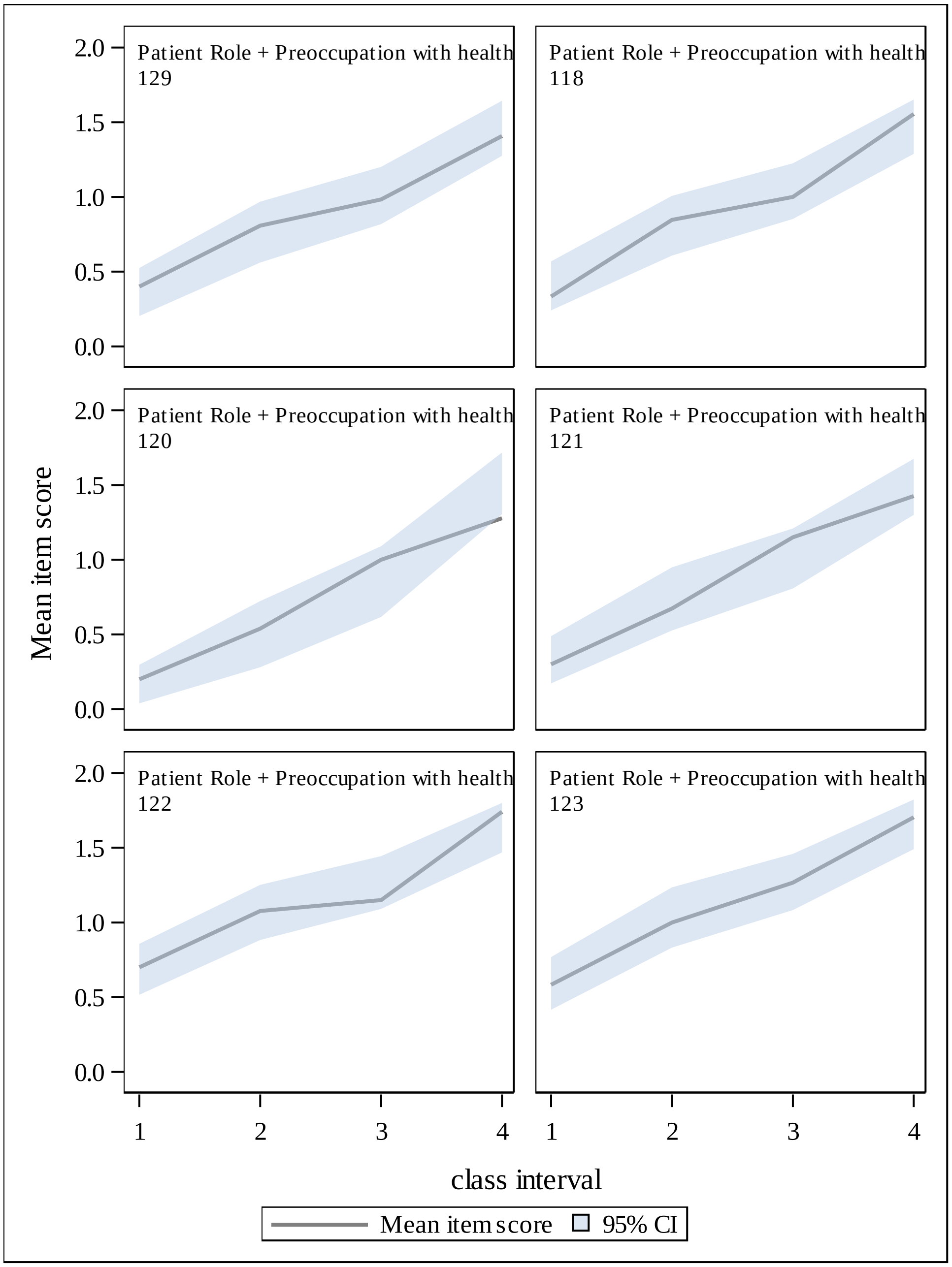

Patient Role + Preoccupation with health 129
Patient Role + Preoccupation with health 118
Patient Role + Preoccupation with health 120
Patient Role + Preoccupation with health 121
Patient Role + Preoccupation with health 122
Patient Role + Preoccupation with health 123
Mean item score
class interval
0.0
0.5
1.0
1.5
2.0
1
2
3
4
Mean item score
95% CI

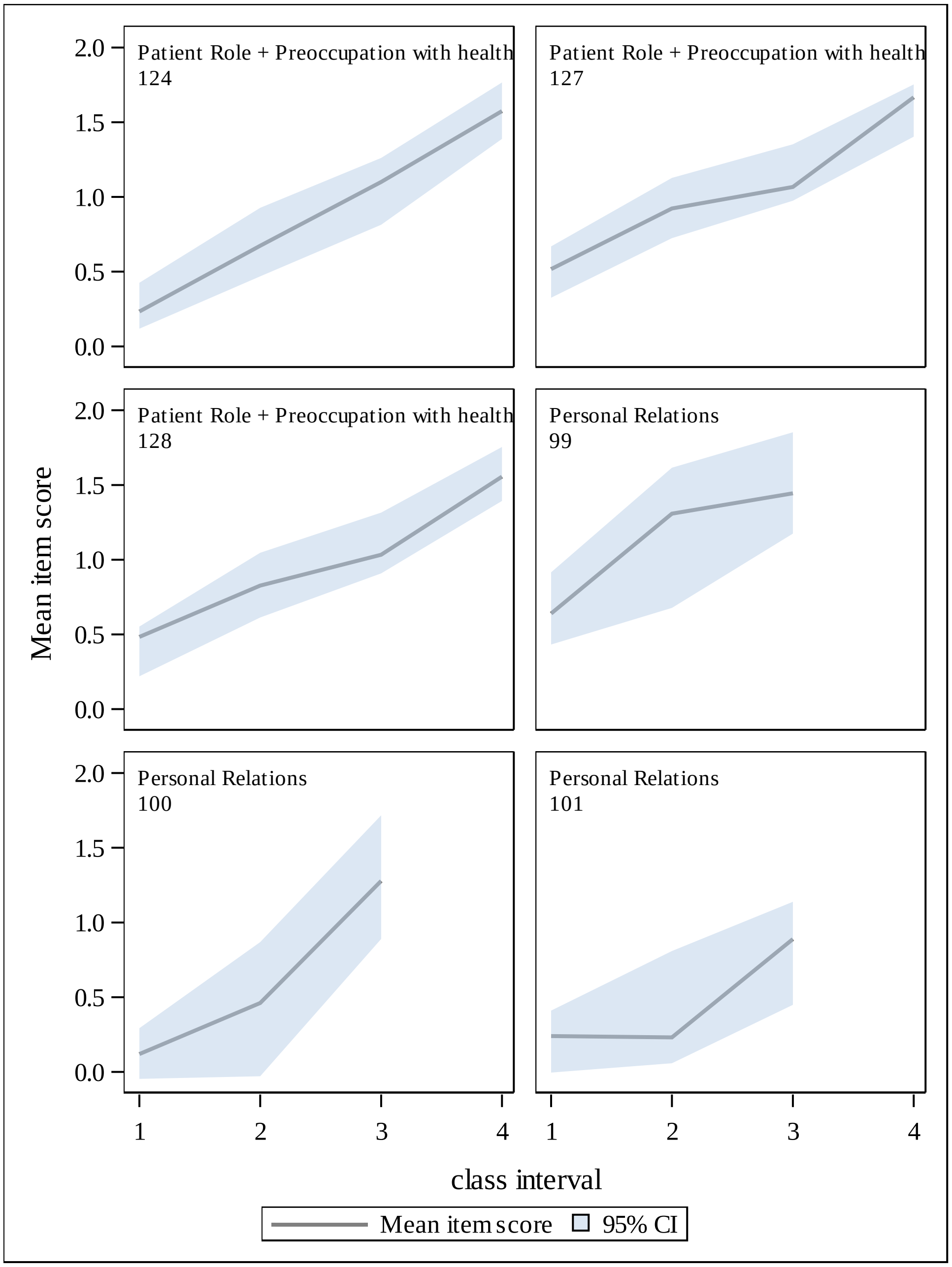
Patient Role + Preoccupation with health
124
Patient Role + Preoccupation with health
127
Patient Role + Preoccupation with health
128
Personal Relations
99
Personal Relations
100
Personal Relations
101
Mean item score
0.0
0.5
1.0
1.5
2.0
1
2
3
4
class interval
Mean item score
95% CI
